# Non-pharmacological interventions for managing fatigue across the lifespan of people living with chronic musculoskeletal conditions: a scoping review

**DOI:** 10.1136/bmjopen-2025-108929

**Published:** 2026-08-04

**Authors:** Katie Fishpool, George Young, Coziana Ciurtin, Fiona Cramp, Emmanuel Erhieyovwe, Bayram Farisogullari, Bethan Jones, Gary Macfarlane, Pedro M Machado, Jennifer Pearson, Eduardo Santos, Emma Dures

**Affiliations:** 1 College of Health and Social Wellbeing, College of Health, Science and Society, University of the West of England, Bristol, UK; 2 Academic Rheumatology, University Hospitals Bristol and Weston NHS Foundation Trust, Bristol, UK; 3 Centre for Adolescent Rheumatology, Department of Ageing, Rheumatology and Regenerative Medicine, University College London Hospital Biomedical Research Centre, London, UK; 4 General Internal Medicine, Hywel Dda University Health Board, Withybush General Hospital Haverfordwest, Carmarthen, UK; 5 Department of Neuromuscular Diseases, University College London, London, UK; 6 Aberdeen Centre for Arthritis and Musculoskeletal Health (Epidemiology Group), University of Aberdeen, Aberdeen, UK; 7 Centre for Rheumatology, Division of Medicine, University College London, London, UK; 8 School of Health, Health Sciences Research Unit: Nursing (UICISA: E), Portugal Centre for Evidence Based Practice (PCEBP), a JBI Centre of Excellence, Polytechnic Institute of Viseu, Viseu, Portugal

**Keywords:** Fatigue, Rheumatology, Paediatric rheumatology

## Abstract

**Abstract:**

**Objectives:**

Fatigue is an important and distressing symptom for many people living with chronic musculoskeletal (MSK) conditions. Non-pharmacological interventions designed to reduce fatigue have been investigated but the evidence is disparate across MSK conditions and across the lifespan. This scoping review aimed to create an overview of the current knowledge of non-pharmacological interventions for fatigue in MSK conditions across the lifespan, including their theoretical bases, the characteristics of participants and the clinical competencies of those delivering interventions. It aimed to use these findings to identify priorities for research and practice in collaboration with patient and public involvement groups.

**Design:**

A scoping review following the Joanna Briggs Institute methodology.

**Data sources:**

AMED, MEDLINE, PsycINFO, CINAHLPlus (EBSCO platform), EMBASE (Ovid platform), SCOPUS and Cochrane databases were searched for studies published between 1 January 2007 and 1 February 2026.

**Eligibility criteria for selecting studies:**

Peer-reviewed primary research studies describing a non-pharmacological intervention with either the intention or effect of reducing MSK fatigue and its impact were included.

**Data extraction and synthesis:**

Title and abstract and subsequent full-text review were performed by two independent reviewers in duplicate. Data were charted in categories according to types of intervention, participant characteristics and theoretical bases. Descriptive analysis of the results is presented.

**Results:**

337 studies met the criteria. Conditions most frequently studied included fibromyalgia, rheumatoid arthritis and systemic lupus erythematosus. Physical activity was the most common intervention type, most participants were adults and approximately 82% were female. 245 studies reported single component interventions, and 80 included a theoretical basis or the clinical competencies required to deliver them. Common exclusion criteria were physical comorbidities, psychiatric disease or unstable health conditions and cognitive impairments.

**Conclusions:**

Few studies have examined how combining single-component interventions can support person-centred fatigue management, and many populations have been excluded from research. To tailor interventions for people with MSK conditions, it is essential to understand how individual characteristics interact with health needs, define the underlying theory, identify the clinical skills required for effective delivery and have more inclusive inclusion criteria.

**STRENGTHS AND LIMITATIONS OF THIS STUDY**

- Patient and Public Involvement and Engagement workshops were held at multiple points during the study, ensuring that the review was informed by lived experience of musculoskeletal-fatigue.
- The scoping review methodology included a broad range of study types to create a map of the available evidence.
- The size and heterogeneity of the review meant that extensive categorisation was required to synthesise data which may lead to a loss of nuanced detail.
- Only evidence available in English has been reviewed.

## Introduction

### Rationale

 Musculoskeletal (MSK) conditions may be inflammatory or non-inflammatory. They include connective tissue diseases, inflammatory arthritis and osteoarthritis, back and neck pain and fibromyalgia and they can affect the muscles, bones, joints and connective tissue.^1 2^ More than 20 million people in the UK and 1.7 billion people worldwide currently live with an MSK condition.^1 2^ Although prevalence increases with age, MSK conditions occur across the lifespan, with an estimated 234 000 children in England and Scotland affected by a chronic MSK condition.^2^

Fatigue has been highlighted by people living with chronic MSK conditions as a priority symptom that substantially affects quality of life and is a challenge to manage.^2–8^ As pharmacological treatments are not licensed for managing fatigue in the absence of active disease, clinical practice has predominantly focused on non-pharmacological approaches.^9 10^ This emphasis is reflected in healthcare research, where recent systematic reviews have evaluated the strength of the evidence for a variety of non-pharmacological interventions across different MSK conditions.^11–15^ There is no consensus about what comprises non-pharmacological interventions; however, for this scoping review, our definition was any sort of intervention not directly involving a medication aimed at preventing, treating or curing health problems.^16 17^

Recent and ongoing studies on the topic of fatigue support the need for this scoping review and have been used to inform its design. A previous scoping review explored reviews of fatigue in adults with rheumatoid arthritis (RA), osteoarthritis, spondylarthritis and fibromyalgia,^18^ reporting on 39 reviews of non-pharmacological interventions, of which 18 addressed fatigue as the primary objective. The authors identified several factors associated with fatigue and a range of interventions, although this was limited to published systematic and narrative reviews and therefore did not include all study designs.

The European Alliance of Associations for Rheumatology (EULAR) recently commissioned a taskforce to evaluate the evidence for non-pharmacological interventions targeting fatigue in inflammatory rheumatic conditions. The resulting systematic review identified several effective approaches, including physical activity and psychoeducational interventions.^14^ These findings informed the development of the EULAR recommendations for the management of fatigue^19^ and underscored the need to further explore contextual factors and the theoretical bases through which these interventions exert their effects.^7^

There is currently no comprehensive understanding of the factors which influence the success of an intervention in reducing MSK fatigue. This has resulted in a lack of evidence to support decision making in how to design, offer and deliver interventions in the most effective way, tailored to a range of patients and at the optimal time. Current clinical guidelines for the management of common MSK conditions recognise fatigue as an important symptom but do not make recommendations for how it can be addressed,^20–22^ hindering implementation of the evidence. Consequently, non-pharmacological interventions for fatigue are not yet part of standard care^23^ and people are not receiving the support they need.

### Review aims and objectives

The review aimed to report existing evidence to inform tailored MSK-fatigue support and to highlight gaps in the evidence base. The review objectives were to explore the type of existing non-pharmacological interventions for MSK fatigue; characteristics of included participants and identify who was missing; the theoretical bases for existing non-pharmacological interventions; the design and outcome measures of the included studies; and location of existing non-pharmacological interventions and the training and skills of those who delivered them.

## Methods

A review protocol was established in accordance with the Joanna Briggs Institute (JBI) methodology for scoping reviews,^24^ supported by two online Patient and Public Involvement and Engagement (PPIE) workshops. These lasted for approximately 1 hour, with discussion facilitated by two researchers (GY and KF) and focusing on people’s experiences of offering or being offered fatigue management support. The participants attending the first workshop included adults living with one or more MSK conditions (n=6) and clinicians from a range of professions who support patients who experience MSK-related fatigue (n=3). The second workshop was attended by children and young people living with MSK conditions (n=12). For the purposes of the literature search and the PPIE meetings, children and young people were defined as those younger than 25 years of age. This age limit was selected to allow for variation in the timing of moving from paediatric to adult rheumatology services and to acknowledge the challenges associated with this transition.^25 26^

Both PPIE groups were presented with the proposed review questions, list of MSK conditions and search terms related to potential intervention types. The participants highlighted additional intervention types and pathways that were subsequently included in the search terms (online supplemental appendix 1). They also deepened the review team’s knowledge of the personal impact of MSK fatigue, confirming it is a significant issue and that an overview of potential interventions would be welcomed by patients and clinicians.

A third, face-to-face workshop was attended by children and young people to explore the data charted from included papers as a pilot of the data charting template presented in the review protocol. This discussion led to amendments to the template which are discussed below.

The review is reported following the Preferred Reporting Items for Systematic Reviews and Meta-Analyses extension for the reporting of Scoping Reviews (PRISMA).^27^

### Systematic literature search

A systematic search of the literature on the AMED, MEDLINE, PsycINFO, CINAHLPlus (EBSCO platform), EMBASE (Ovid platform), SCOPUS and Cochrane databases was performed in November 2023 and citation searching was completed in February 2024, following the published review protocol.^28^ The same searches were re-run in February 2026 to enable the inclusion of studies published between 1 November 2023 and 1 February 2026. Only studies published in the English language were included due to a lack of resources to support translations.

### Study selection

Records were imported into Covidence and duplicates were removed. Each abstract was screened by two or more independent reviewers (KF, GY, CC, BJ, ED and EE) and compared against the eligibility criteria (table 1) to determine inclusion. Discrepancies were checked and resolved by discussion (KF, GY and ED). At full-text screening stage, all papers were screened by two independent reviewers (KF and GY).

**Table 1:** Eligibility criteria

Inclusion criteria	Exclusion criteria
Primary research study Published in a peer-reviewed journal Available in English language Participants have one or more chronic MSK conditions Participants experience fatigue at baseline Published during or after 2007 Describes a non-pharmacological intervention to manage MSK condition symptoms, with fatigue reduction as a primary or secondary outcome	Reviews, protocols, opinion pieces, editorials, case series, case reports, observational cohort studies Pharmacological interventions No intervention is described Muscle fatigue rather than global fatigue is examined No data are available on factors associated with intervention success (theoretical basis of intervention or characteristics of participants or characteristics of practitioners delivering interventions)

MSK, musculoskeletal.

### Data charting

Data were sought to address each of the review objectives and included the MSK condition(s) being studied, the number of participants included, demographic details of the participants and reasons for exclusion from participation, the tools used to measure fatigue, details of the studied interventions and their theoretical bases. These data were extracted from the included papers by two reviewers (KF and GY), using a template^29^ to ensure consistent recording (online supplemental appendix 2). Double-checking of extracted data was completed for 85% of included articles to ensure accuracy. Disagreements were resolved by consensus, with an additional reviewer (ED).

The extracted data were analysed using descriptive, narrative methods due to the heterogeneity of the included studies.^30^ This involved frequency counts and basic qualitative content analysis. KF and GY familiarised themselves with the data, used open coding and organised codes into categories. These categories were discussed with the wider study team. In keeping with JBI guidance, reinterpretation of the original study data was avoided.

### Deviations from the protocol

During the screening process, the definition of fatigue as a primary outcome was reviewed by the study team to ensure consistency. Due to the heterogeneity of included studies, fatigue was considered the primary focus if this was explicitly stated, if a fatigue measurement tool had been used to determine sample size or if a hypothesis for how the intervention would act on fatigue was provided. The rationale for this was to identify the conceptual understandings of MSK fatigue and how they informed the research and approaches to fatigue management. It was also agreed that where multiple papers referred to the same study, these would be grouped together into a single record to remove duplication while ensuring that any rich contextual data were captured.

The data charting template presented in the review protocol was piloted by presenting the initial findings to a PPIE workshop group. In March 2024, a group of children and young people (n=7) reviewed this initial data and shared their opinions on which elements mattered to them and what was missing. This highlighted several elements of interventions which participants felt were important to ensuring safety but were not being captured.

Amendments to the data charting template were made to address this, recognising the importance of physical safety, in the sense that those involved in delivery were appropriately qualified and experienced in MSK health, and psychological safety, in the sense that those involved in delivery created an environment in which participants felt accepted and supported. The additional items included in the data extraction template included: (1) the knowledge and experience of the facilitator specific to the intervention, (2) the level of supervision of the intervention and (3) the physical and social environment where the intervention was delivered (online supplemental appendix 2).

### Patient and public involvement

PPIE is a key aspect in designing research for real-world impact.^22^ The purpose of a scoping review was to understand the broad overview of the existing evidence, including what was missing. PPIE is a vital part of this process and their insights and priorities helped to make sense of large heterogeneous data sets and how the evidence related to their lived experience.^23^

We have reported the outcome of PPIE events and how they influenced the scoping review process following the guidelines for the GRIPP2 short form reporting checklist,^24^ a tool designed to improve the reporting of PPIE in research (online supplemental appendix 3).

## Results

**Figure 1:**
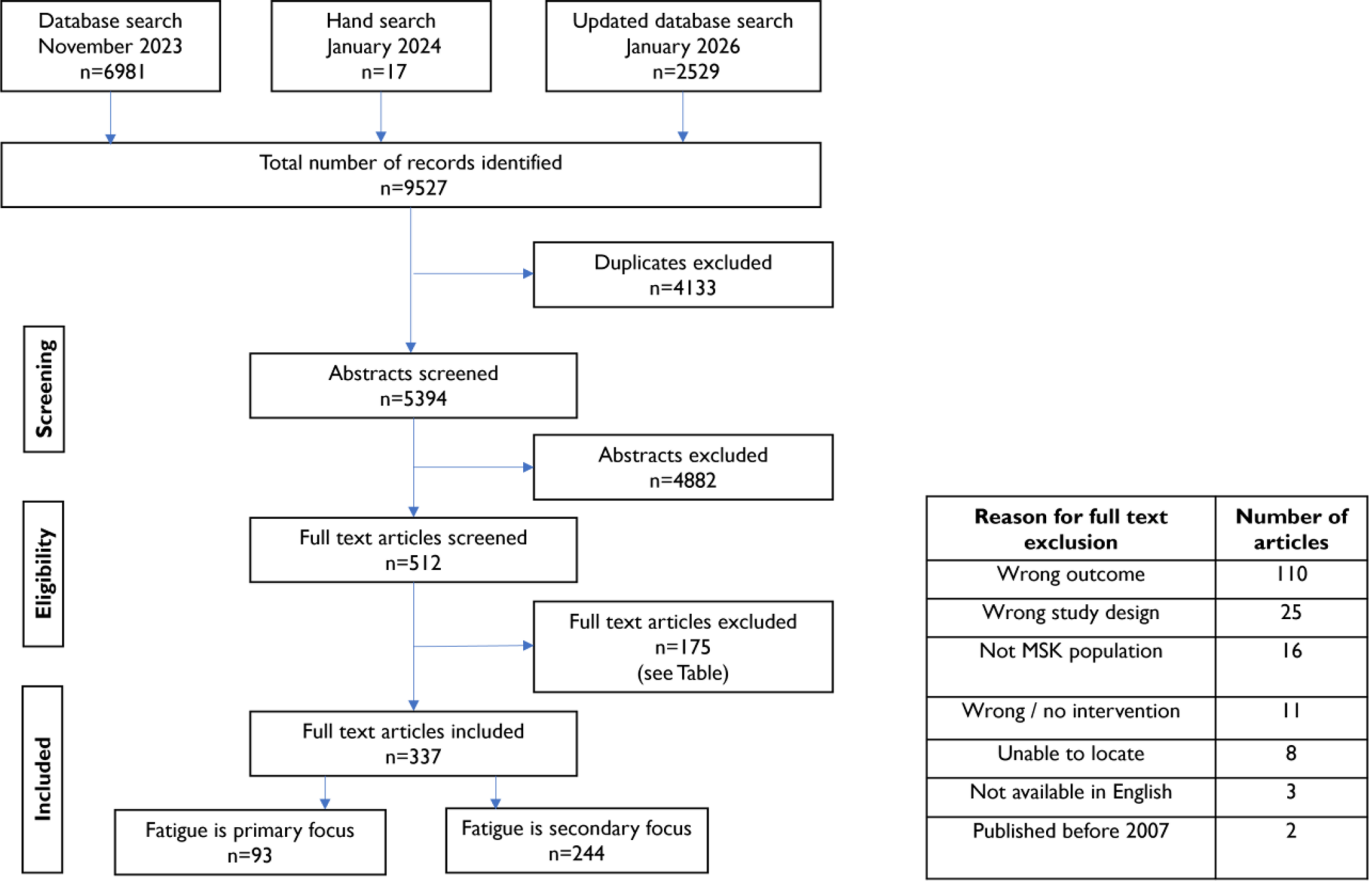
PRISMA flow diagram. MSK, musculoskeletal; PRISMA, Preferred Reporting Items for Systematic Reviews and Meta-Analyses.

The screening process is reported using a PRISMA flow diagram (figure 1), including a tabulated summary of reasons for exclusion at full text screening.

### Review objective 1: explore the type of existing interventions

#### Participant numbers and intervention types

The evidence search found 337 studies where the impact of an intervention on fatigue was measured. In the 93 studies which named fatigue as the main outcome of interest, there were a total of 9465 participants, compared with 22 702 participants in the 244 studies where fatigue was captured as a co-existing symptom or incidental finding. In studies where fatigue was of secondary interest, the most common primary focus was pain.

The types of intervention explored were categorised as educational, nutritional, passive therapy, physical activity, psychological or multicomponent (table 2). A description of individual interventions can be found in online supplemental appendix 4.

**Table 2:** Definitions of intervention types

Intervention type	Definition
Educational	Interventions designed to increase knowledge and understanding of a health condition, specific symptoms or self-management skills.
Nutritional	Interventions that included dietary changes or supplements taken orally.
Physical activity	Interventions based on intentional movement or exercise.
Passive therapy	Interventions provided by a device or practitioner that did not require physical or mental exertion by the participant, for example, massage, acupuncture.
Psychological	Interventions designed to address thoughts, feelings and patterns of behaviour.
Multicomponent	Including two or more non-pharmacological interventions.

Most included studies, with fatigue as either primary or secondary focus, comprised a single category of intervention; however, 92 studies reported on the use of multicomponent interventions (table 3). The most common elements of multicomponent interventions were physical activity, education and psychological support.

**Table 3:** Intervention types and participant numbers from included studies

	Studies with a primary focus on fatigue (participants)		All included studies (participants)	
	Single component	Multicomponent	Single component	Multicomponent
Physical activity	32 (2659)	17 (2067)	113 (7160)	75 (8312)
Passive therapy	20 (1907)	4 (327)	58 (6837)	26 (2711)
Psychological	9 (784)	13 (1405)	35 (3092)	47 (5852)
Educational	8 (1659)	12 (1184)	24 (4098)	61 (7172)
Nutritional	3 (155)	3 (209)	15 (703)	11 (971)
Total	73 (7164)	22 (2303)*	245 (21 890)	92 (10 002)*

* The total number of multicomponent interventions is fewer than the individual count for types of intervention included, as a single study may cover several individual elements.

The number of participants varied significantly with multicomponent studies tending to be larger than those reporting on a single component intervention. Physical activity interventions were the most studied when fatigue reduction was either the primary or secondary focus and regardless of whether a single or multicomponent intervention was being reported. Both passive therapeutic interventions and psychological interventions were included in many studies of single interventions; however, passive therapeutic interventions appeared less frequently as part of a multicomponent study. Educational interventions were the basis of only a small number of single component studies but appeared frequently in multicomponent studies. Nutritional interventions were reported in fewer studies than any other type of intervention and had fewer participants.

#### Changes over time in the evidence being collected

There has been a notable increase in the number of studies that measure fatigue since 2007. Fatigue as a primary focus of studies has also increased but at a slower rate. There has been an increase in the study of multicomponent interventions, though investigations of single component interventions remain the most common. There has also been an increase in the frequency of studies including a nutritional component, although nutritional interventions remain the least common overall.

#### Geographical location of studies

The 337 included studies originated from 45 different countries across 6 continents. Most studies had a European origin (n=198) and the countries associated with the greatest number of eligible studies were the USA (n=57), Turkey (n=47) and Spain (n=32).

### Review objective 2: explore the characteristics of participants and identify who is missing

#### Sex

**Figure 2:**
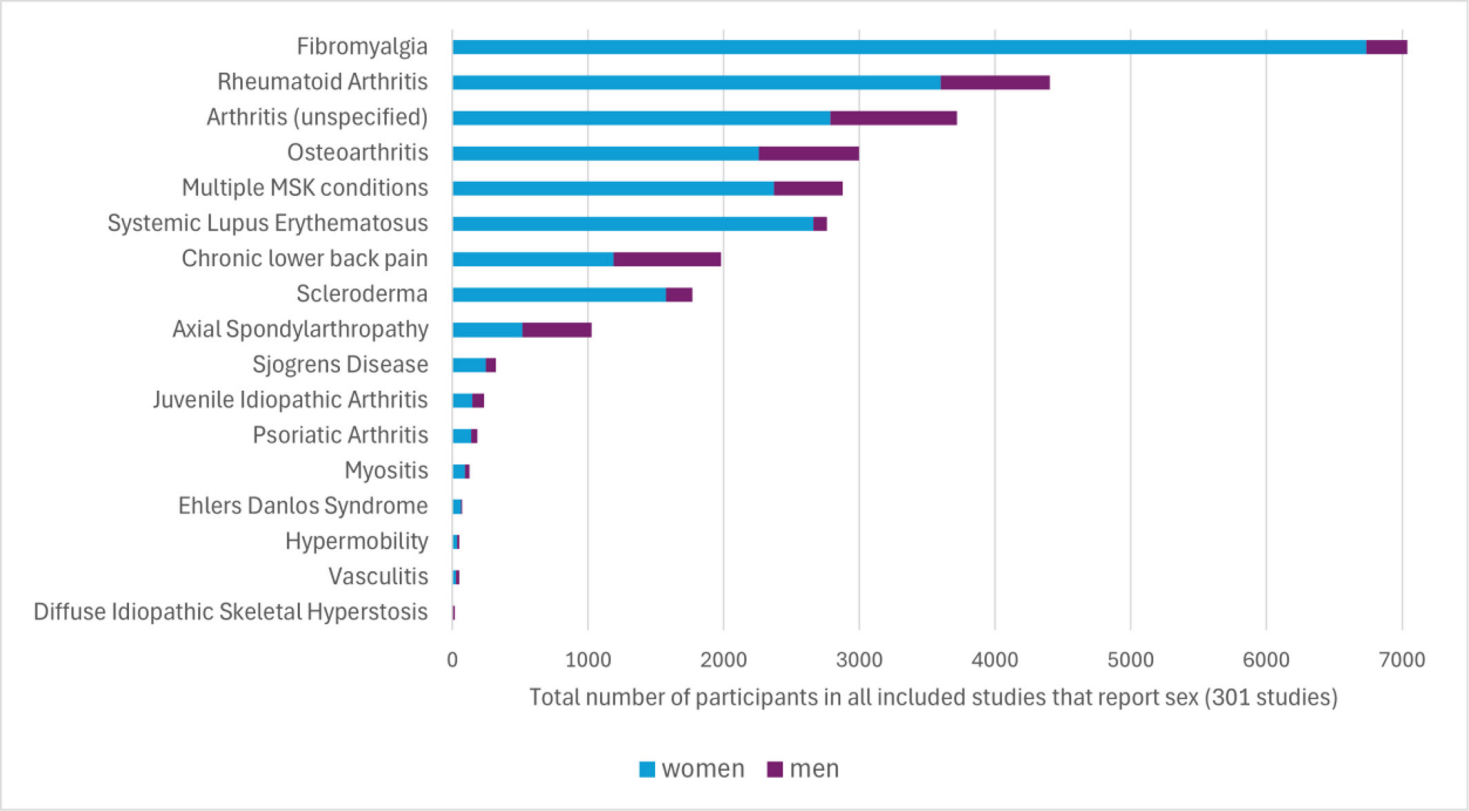
Sex of participants included in studies. MSK, musculoskeletal.

The sex of participants was reported in 301 of the included studies, of which 79 focused primarily on fatigue. Approximately 18% of research participants were men; however, the spread of participants by sex was uneven and skewed when viewed either by the intervention type or condition. Men were in the minority in all intervention types, most notably in nutrition, education and psychology (Figure 2).

103 studies only recruited women, no studies recruited exclusively men and only 19 of the 301 studies that reported data on sex had a majority of men taking part. Men have approximately equal representation in the research data relating to axial spondylarthritis (AxSpA), chronic lower back pain (CLBP), vasculitis and diffuse idiopathic skeletal hyperostosis.

In studies relating to fibromyalgia, systemic lupus erythematosus (SLE), Ehlers-Danlos Syndrome and scleroderma, only a very small number of men were included, with many studies recruiting only women and some excluding men from participating.

#### Life stage of participants

The age of participants was reported in 329 of the included studies. Most studies included only adult participants (those aged 18 years or over), with 27 stratifying their results so findings relating to older adults (those aged over 65 years) could be identified and a further seven including exclusively older adults. Two studies included both adult and child participants and a further 16 reported only on children and young people. In all age groups, physical activity was the most explored intervention.

#### Clinical and demographic data

**Figure 3:**
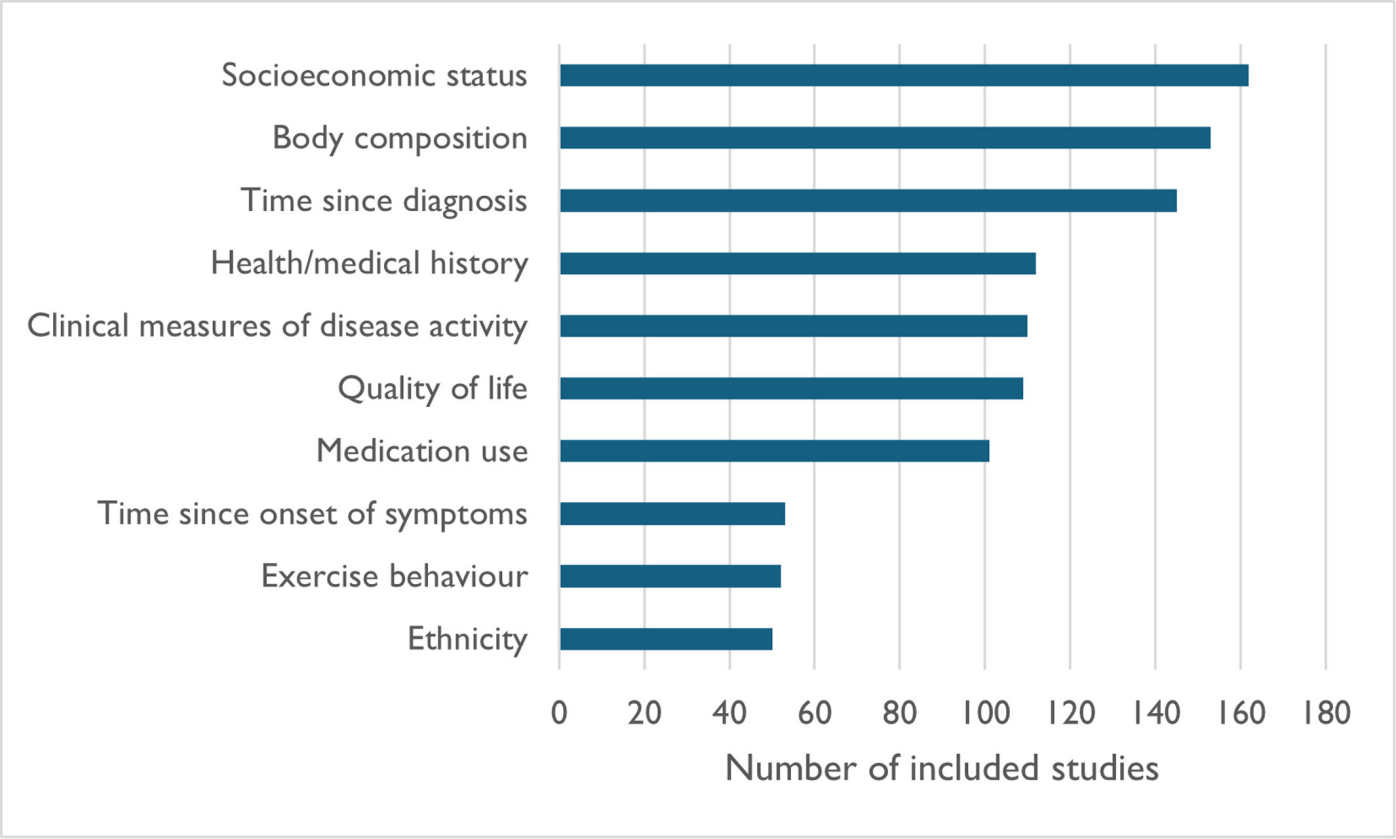
Frequency of collection of sociodemographic data.

Clinical and demographic data of participants were collected in 296 of the included studies (figure 3). The data collected most frequently were the socioeconomic status of study participants (n=162). Several studies discussed the impact of wider determinants on health literacy, patient activation and access to resources. Most participants were from medium or higher income households and had a higher than average level of formal education.

Data on body composition (height, weight, body mass index (BMI)) were collected in almost half of the included studies (n=153). In most cases, the reasoning for this was not explained in the study, but a minority discussed the role of overweight and obesity in contributing to inflammation, potentially worsening fatigue.

Data on the time since diagnosis were also collected in 145 studies, which was noticeably distinct from time since onset of symptoms. Those studies which collected data on both often stated the impact of delayed diagnosis on the physical and mental well-being of patients.

It is significant that ethnicity data were collected in a relatively small number of studies (n=50), as there is evidence that some ethnic groups are underrepresented in research and also that cultural sensitivity is needed to ensure that interventions are acceptable to the target population.^31^ In the studies that did collect data about ethnicity, most participants were white Europeans.

#### Exclusion criteria

**Figure 4:**
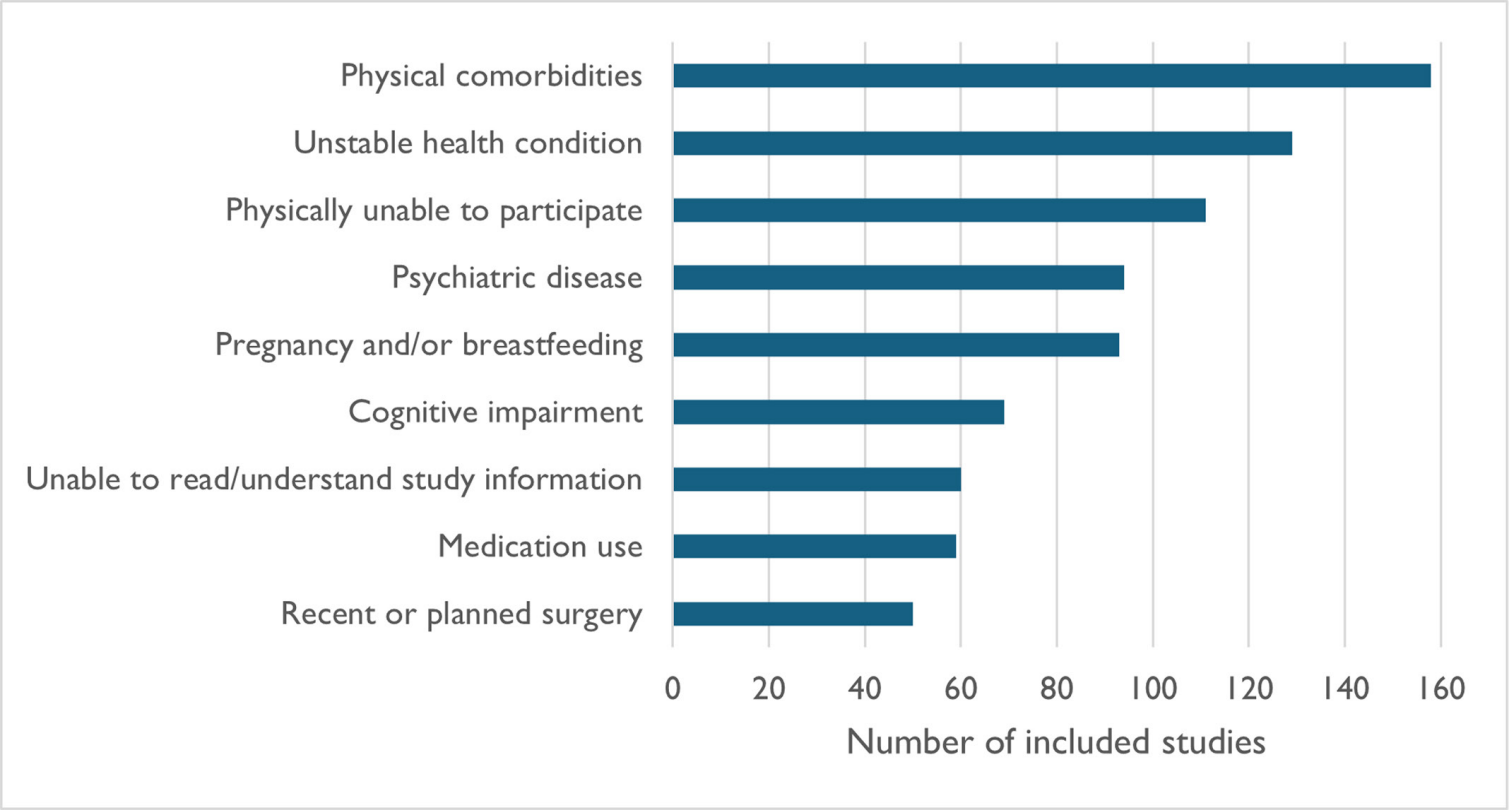
Frequency of recorded exclusion criteria.

The criteria for excluding people from participation were reported by 268 included studies (Figure 4). This was explored to understand who is not represented in the current evidence base. The most common exclusion criteria were physical comorbidities or unstable health conditions reported in almost half of the included studies. Pregnancy and breastfeeding were also common reasons for excluding people, which is significant because most participants were women and many of the frequently studied conditions such as SLE, RA and fibromyalgia are prevalent in women of childbearing age.

Many studies excluded people who were living with cognitive impairment (n=69) or unable to read/understand the study information (n=60). There are many reasons why a person may not be able to understand given information such as spoken languages, health literacy or disability; however, the nature of this was rarely explored.

Other exclusion criteria included a history of substance misuse (n=16) or BMI either above (n=7) or below (n=4) a stated value.

### Review objective 3: explore the theoretical basis for interventions

The theoretical basis included information on how the intervention was hypothesised to work. This information might also include mechanisms of action which described the specific processes through which the intervention produced its effects. This was stated in 80 of the included studies, including 36 studies where the aim of the intervention was primarily to reduce fatigue (online supplemental appendix 5). The stated theories were categorised as being biochemical, physical and/or psychological in nature (table 4). Most interventions (n=68) were based on a single type of theory; however, some studies were based on two distinct types of theory, with several mechanisms of action for reducing fatigue arising from their intervention.

**Table 4:** The type of theoretical basis in relation to the category of intervention

Theoretical basis	Included studies*	Intervention category				
		Educational	Nutritional	Passive therapy	Physical activity	Psychological
Biochemical	38	5	8	20	16	4
Physical	31	1	2	10	20	4
Psychological	23	9	1	1	13	11

* The total number of included studies is fewer than the combined individual counts across intervention type where an intervention has included multiple components.

A biochemical theoretical basis was described in 38 studies, most often in condition-specific interventions such as fibromyalgia (n=21). Potential mechanisms discussed included stimulation of the endocrine system by increasing neurotransmitter activity, regulating cortisol levels, reducing oxidative stress and regulating metabolic activity. Other studies considered improvement of microcirculation, sympathetic nervous system regulation, immune system support, management of the gut microbiome and reduction of inflammation, specifically reducing obesity-induced inflammation, as potential biochemical mechanisms.

A physical theoretical basis was most frequently explored in studies of physical activity interventions. Potential mechanisms included muscle relaxation, pain reduction, improved sleep quality, reduced BMI, increased aerobic capacity and increased muscle strength. Many conditions were represented in these studies, including CLBP, RA, AxSpA, SLE, psoriatic arthritis, juvenile idiopathic arthritis, fibromyalgia, scleroderma, Sjögren’s disease, myositis and hypermobility.

A psychological theoretical basis was explored in 23 studies. Potential mechanisms included internal cognitive, emotional and motivational processes, increased knowledge about fatigue in the context of an MSK condition, peer support, validation, increased self-efficacy, reduction of stress, acceptance and fear reduction. Often, these mechanisms were intended to bring about changes in health behaviour, for example, using activity pacing.

### Review objective 4: explore the design and outcome measures used

In total, 337 records of unique studies were included in this review, of which 93 primarily focused on fatigue. The predominant methodology was quantitative (n=320) and the remaining studies used mixed methods (n=17). Where quantitative studies were published alongside one or more complementary qualitative papers these associated papers were grouped as single studies and data extracted from them accordingly, to avoid duplication while ensuring that rich contextual data were captured.

#### Fatigue measurement tools

A total of 35 tools were used to measure fatigue in the included studies, with 24 measurement tools used to assess those interventions specifically intended to reduce MSK fatigue (online supplemental appendix 6). 15 tools were identified that were specific to a single condition and 20 were validated for use in a range of health conditions. An age-specific tool, the Paediatric Quality of Life, was used to measure fatigue in eight of the 16 studies that included children and young people.

### Review objective 5: explore the location of interventions and the training and skills of those who delivered them

#### Location of delivery

The location where interventions were delivered was reported in 232 of the included studies. The most frequently reported locations were hospital outpatient clinics (n=86) and participant homes (n=81), with a minority (n=30) being delivered across multiple locations. Interventions were delivered both virtually and in person, with online delivery, telephone calls or text messages being used in 55 studies. Outside of hospitals, interventions were delivered in healthcare or education settings such as general practitioner practices, private health clinics and university departments (n=30) and in community settings including gyms, swimming pools, community buildings and outdoor spaces (n=27). Two studies reported on interventions delivered in the participants’ workplaces.

#### Intervention facilitators

Interventions were delivered by healthcare professionals, complementary medicine practitioners, physical activity instructors, researchers and peer support networks. The profession or role of the intervention facilitators was reported in 229 of the included studies and the majority of these (n=170) were delivered by one or more healthcare professionals.

**Figure 5:**
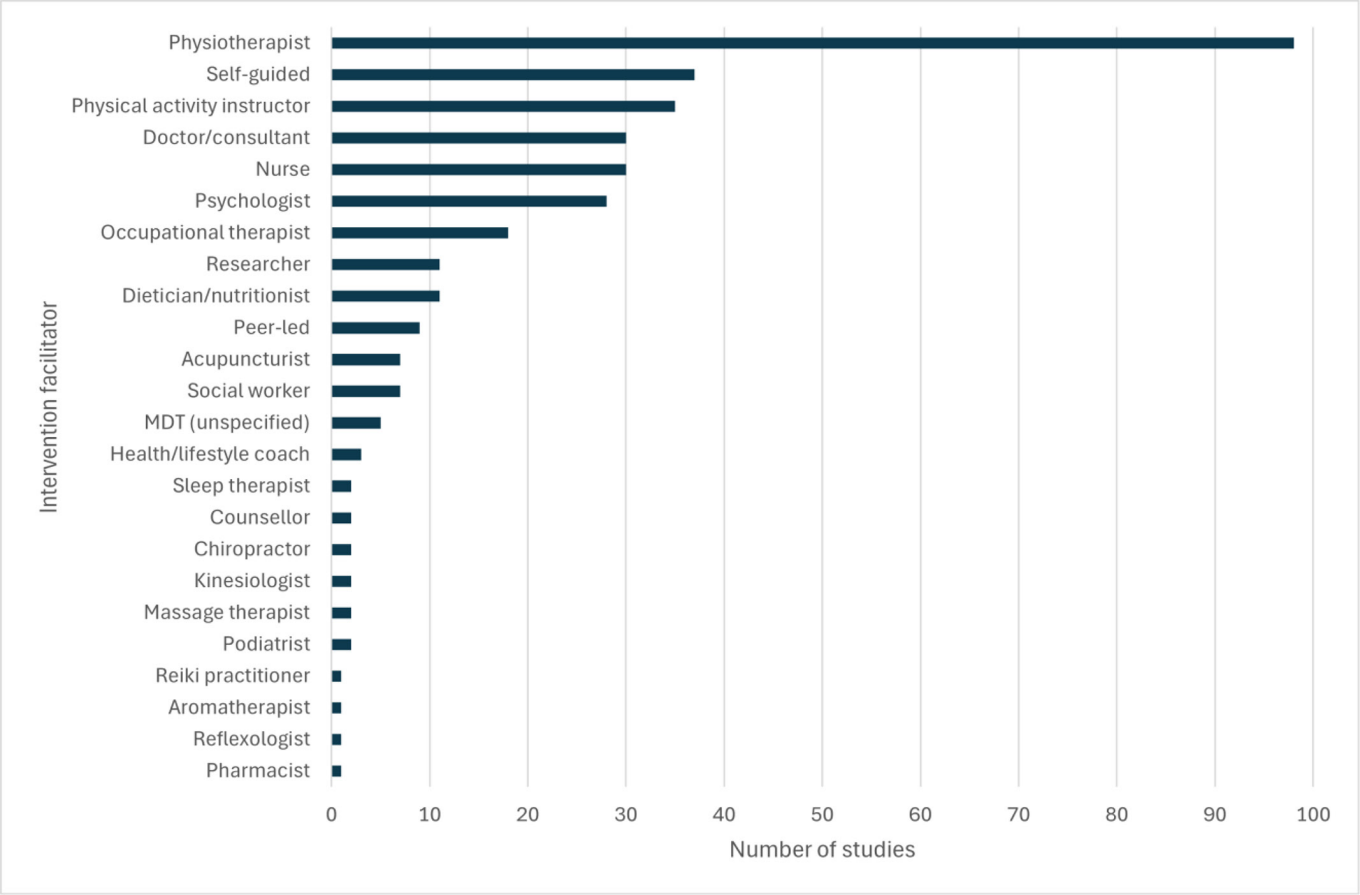
Role or profession of people facilitating interventions. *Multidisciplinary team (MDT)

In addition to details of the profession or experience of the intervention facilitators, 64 studies provided further details on the specific training which those delivering the intervention completed to support their role. Most interventions were delivered in a group format, although a notable minority (n=37) were self-guided (see figure 5).

## Discussion

This scoping review identified many studies examining the impact of non-pharmacological interventions on fatigue in MSK conditions. There has been an increase over recent years, which may partly reflect the inclusion of fatigue as a core outcome following the eighth OMERACT meeting.^32^ However, fatigue often remains a secondary outcome rather than the primary target of intervention.

The MSK conditions represented in the included studies were broadly reflective of their prevalence in the general population.^33^ Fibromyalgia was the most frequently studied condition, and studies in this area most often described mechanisms of action. This may indicate the prioritisation of fatigue as a key symptom in this population, or a greater emphasis on non-pharmacological management in line with EULAR recommendations compared with other MSK conditions.^34 35^

Physical activity was the most studied intervention, both as a standalone treatment and as part of multicomponent approaches. While its efficacy and safety for fatigue management are well established^14^ and it is one of the EULAR recommendations,^19^ individuals with MSK conditions are less likely to engage in physical activity than the general population.^36 37^ Nutrition was the least studied intervention type, appearing in only 26 studies, despite being an area of strong interest among PPIE contributors across age groups. Consequently, there is a lack of clinical guidance and PPIE contributors often relied on informal sources such as peer groups or social media. Interest in nutrition may stem from the perception that it is something within people’s control that can be integrated into daily life. However, methodological challenges, including accurate dietary measurement and confounding factors, complicate the design of high-quality nutritional research.^38^ Although the number of studies in this area is increasing, further research and clearer clinical guidance are needed.

The biochemical, physical and psychological theoretical bases underpinning interventions in the scoping review highlight the multifaceted nature of fatigue. Although a minority of studies provided detailed descriptions, the diversity of proposed mechanisms of action reflects multiple approaches to managing fatigue and the need for tailored strategies.^18^ The increasing frequency of multicomponent interventions suggests a growing recognition of the complexity of the symptom of fatigue. A specific approach mentioned by PPIE contributors as helpful was activity pacing, a goal-directed behavioural strategy that helps individuals manage energy across daily activities.^39^ Despite its potential to benefit people living with MSK fatigue, only four included studies focused on pacing, typically as part of broader educational interventions. This limited evidence base is reflected in a recent meta-analysis, which identified only six relevant studies across fatigue-related conditions, with just two including MSK populations.^40–42^

Most interventions were delivered in clinical settings by healthcare professionals. Although cost data were not collected, differences in cost-effectiveness across intervention types are likely,^43^ which has implications for feasibility and sustainability. There remains a lack of evidence on the cost-effectiveness of non-pharmacological interventions, which is necessary for determining optimal models of fatigue management.^14^

Participant characteristics were inconsistently reported in the included studies. Socioeconomic status and body composition were frequently collected, possibly reflecting recognition of health disparities in MSK conditions. In contrast, ethnicity was rarely reported, and where it was, participants were predominantly White Europeans. This is notable given evidence from England showing that individuals of Pakistani or Black Caribbean heritage are disproportionately affected by MSK conditions,^44^ and that some ethnic groups are less likely to achieve disease remission within 3 months of treatment.^45^ A better understanding of the broader determinants of MSK health is needed to ensure interventions meet the needs of those most affected by fatigue.

Exclusion criteria in the included studies are likely to have limited the representativeness of the evidence. Individuals with physical comorbidities, psychiatric conditions, cognitive impairments, or those who were pregnant or breastfeeding were often excluded. As non-pharmacological interventions are likely to benefit these populations, it is important that future research includes them and considers their specific needs. Addressing barriers to implementation will also be critical in narrowing the evidence-to-practice gap. Sex imbalances were also observed across studies. While some of this may reflect underlying condition prevalence, it does not fully explain the disparity. This is important given evidence that men and women differ in psychological responses, coping strategies and support preferences.^46^ There was also limited research focused specifically on children and older adults, despite differing needs across the lifespan. This was highlighted by young people in PPIE workshops, who emphasised the interplay between education, health and social life when considering intervention suitability.

A strength of the scoping review was the involvement of PPIE contributors throughout, ensuring the research aims and objectives aligned with patient priorities. PPIE contributors influenced the search strategy, data extraction, data interpretation and prioritisation of the findings. Discussions included the need for accessible, reliable information and the importance of offering a range of fatigue management options within routine rheumatology care. Another strength was the methodology which enabled inclusion of a broad range of study designs, providing a comprehensive overview of the evidence base and identifying areas of overlap and gaps.^47^ However, synthesising such a large and heterogeneous body of literature required extensive categorisation, which may have resulted in some loss of nuance. For example, exercise interventions may have incorporated psychological components that were not fully captured. Additionally, studies were not critically appraised. Finally, the exclusion of non-English language studies may have limited the diversity of perspectives and participant populations.^48^ As most included studies originated from Europe and North America, findings are dominated by Western healthcare perspectives. Including a wider range of languages and databases could reveal additional evidence, particularly in currently underrepresented areas.

## Conclusions

Fatigue is frequently considered in studies of symptom management for MSK conditions. The causes and experience of fatigue are widely acknowledged as multidimensional and a wide variety of theoretical bases and mechanisms of action for reducing fatigue are proposed in the literature. There are relatively few studies which explore how approaches can be combined to achieve person-centred fatigue management, although studies of multicomponent interventions are becoming more common.

Physical activity interventions were the most widely studied but more research is needed to understand how these interventions can be made accessible and acceptable for those groups who are living with highly intrusive symptoms of fatigue. The involvement of the PPIE groups in exploring the review findings has demonstrated a potential disconnect between the priorities of patients and those of researchers in terms of preference for intervention types and for information-sharing needs.

Gaps in the evidence base have been identified, including limited evidence on appropriate interventions for managing fatigue in children and young people. While the theoretical bases and mechanisms of action of some interventions may be similar across the lifespan, the criteria which determine whether an intervention is accessible and acceptable may vary across the lifespan. Future studies should also explore participants’ perceptions in relation to their physical and psychological safety.

Other under-represented groups included men, people with complex or unstable health conditions and those living with cognitive or intellectual impairment. To effectively tailor interventions to the needs of individuals, it is important to understand how these characteristics may interact with health needs.

## Supplementary material

10.1136/bmjopen-2025-108929 online supplemental appendix 1

10.1136/bmjopen-2025-108929 online supplemental appendix 2

10.1136/bmjopen-2025-108929 online supplemental appendix 3

10.1136/bmjopen-2025-108929 online supplemental appendix 4

10.1136/bmjopen-2025-108929 online supplemental appendix 5

10.1136/bmjopen-2025-108929 online supplemental appendix 6

## Data Availability

Data are available on reasonable request.
