# Supplementary material for: Non-pharmacological interventions for managing fatigue across the lifespan of people living with chronic musculoskeletal conditions: a scoping review
Source: BMJ Open. 2026 Aug 4;16(8):e108929. doi: 10.1136/bmjopen-2025-108929 (PMC13448569; doi:10.1136/bmjopen-2025-108929)
Supplement: Supplementary file 1 [file bmjopen-2025-108929.reviewer_comments.pdf]

PEER REVIEW HISTORY

BMJ Open publishes all reviews undertaken for accepted manuscripts. Reviewers are asked to complete a checklist review form and are provided with free text boxes to elaborate on their assessment. These free text comments are reproduced below.

ARTICLE DETAILS

Title (Provisional)

Non-pharmacological interventions for managing fatigue across the lifespan of people living with chronic musculoskeletal conditions; a scoping review

Authors

Fishpool, Katie; Young, George; Ciurtin, Coziana; Cramp, Fiona; Erhieyovwe, Emmanuel; Farisogullari , Bayram; Jones, Bethan; Macfarlane, Gary; Machado, Pedro M.; Pearson, Jennifer; Santos, Eduardo; Dures, Emma

---

VERSION 1 - REVIEW

Reviewer 1

Name Antcliff, Deborah

Affiliation Northern Care Alliance NHS Foundation Trust, Bury Integrated Pain Service

Date 29-Aug-2025

COI None

Thank you for the invitation to review the manuscript entitled ‘A scoping review of non-pharmacological interventions for managing fatigue across the lifespan of people living with chronic musculoskeletal conditions’. This has the potential to be an interesting scoping review, but at present, some areas are difficult to follow and lack detail and clarification. I wondered if BMJ Open could clarify how recently they request systematic literature searches to have been undertaken and whether Nov 2023 is acceptable; and also for BMJ Open to clarify their permissive use of identical text from a published protocol in the findings paper written by the same author (see the Introduction, first four paragraphs).

Please also see major suggestions below:

Title

I noted the authors had changed the current title from the published protocol: “Factors influencing the outcomes of nonpharmacological interventions for managing fatigue across the lifespan of people living with musculoskeletal (MSK) conditions: a scoping review protocol”. I think the current title is perhaps more suitable since it cannot be assumed that

the factors identified (e.g. sample population, theoretical basis, clinical expertise etc) directly influenced the outcomes, without undertaking specific study into each factor. However, does this change in title lessen the link between the protocol and the current paper. Do the authors wish to add 'factors' somewhere in the present title?

Abstract

i) As per the PRISMA-ScR checklist, the abstract may benefit from comprising: background, objectives, eligibility criteria, sources of evidence, charting methods, results, and conclusions. At present, the abstract is missing clear eligibility criteria (e.g. types of evidence such as RCTs, cross-sectional studies etc), sources of evidence (e.g. databases etc) and charting methods.

ii) Objectives: in the sentence "to highlight gaps and collaborate with Patient and Public Involvement groups to identify priorities", please add information on what priorities you are seeking to identify.

iii) Results – I am unclear on the sentence: "Two hundred and sixty-eight eligible studies were found, including 72 studies primarily focused on fatigue." From reading the start of the abstract, it would seem that a primary focus on fatigue would be part of the inclusion criteria. Please clarify here your meaning, e.g. fatigue as a primary outcome compared with other included studies in which fatigue was a secondary outcome (if that is the case). That is, please clarify how fatigue played a role in the papers that were included in which fatigue was not the primary focus.

iv) Results: in the sentence "Conditions most frequently studied included fibromyalgia, rheumatoid arthritis and systemic lupus erythematous", could any percentages be provided of the proportion of studies that included the above conditions.

v) Results – could a further sentence be added to report on some key findings linked to all of the aims of the review, e.g. any identified theoretical basis of the interventions, clinical competencies of those delivering interventions etc. Please also see latter comments on the use of 'theoretical basis', and whether 'mechanism' is more appropriate (as per Appendix 5).

vi) Please consider providing a further sentence in the Results to link with the first sentence of the Conclusion: "Few studies explore how interventions can be combined to achieve person-centred fatigue management", since the Results do not specifically mention using singular treatments. This sentence feels out of context at present.

Introduction

i) Rationale: the first, second and fourth paragraphs contains identical text (and same references) as per the author's published protocol (with similar content also in paragraph 3) (https://bmjopen.bmj.com/content/bmjopen/14/5/e082555.full.pdf). Please check with the journal whether this exact replication of text is permissible.

ii) Please check the number of people living in the UK with chronic MSK conditions since the published protocol states 10 million, but the present manuscript states 20 million. The same references have been used in both. However, these are webpages and so the number may have changed over the last 2 years. Please clarify whether the number is 10 or 20 million and please add the date the website was visited to the References section.

iii) Please consider re-locating the PPI statement in the Methods rather than at the end of the Introduction.

Methods

i) Line 25-26: "A systematic search of the literature was performed in November 2023 and citation searching was completed in February 2024". Have the searches been re-run to check for any new eligible papers that have been published in the last 1-2 years?

ii) Page 6, line 6: the authors define their meaning of fatigue being a primary focus. However, they appear to include papers in which fatigue is not the primary focus. Please clarify how fatigue plays a role in such papers, and why they were still included in a review that focuses on fatigue.

iii) Please can the authors expand on the analysis section of the Methods, beyond stating narrative methods were used (e.g. actual analysis such as coding/thematic analysis, methods to present the results, methods to summarise/synthesise the evidence). If these stages did not occur, please state as such and explain why.

iv) Please can the authors state they have reported the review as per the PRISMA-ScR checklist in the Methods.

v) As per the PRISMA-ScR checklist, please can the authors add detail to the Methods to "list and define all variables for which data were sought and any assumptions and simplifications made." For example, the authors state in the objectives they will explore papers for theoretical basis, which is not mentioned in the Methods.

vi) Were any critical appraisals undertaken of the included studies? If not, please can this be stated as a limitation in the Discussion.

vii) Please signpost to the PRISMA flowchart from the Results not the Methods.

Results

i) Please commence the results with the details as per the PRISMA flowchart to report on number of studies identified and included in the review. I am unclear as to which studies were included or excluded. Specifically, the authors describe papers in which the primary focus is on fatigue. If the primary focus was not on fatigue, were the papers still included in the review? Please clarify your meaning of fatigue being a primary focus, e.g. as a primary outcome, compared with other included studies in which fatigue was a secondary outcome (if I have interpreted this is correctly). See earlier comment on this in the Methods.

ii) Page 7, line 21: “the studies which focused primarily on fatigue had smaller numbers of participants.” Please see point Results, comment i) about clarifying which papers you have included as being focused on fatigue. Please also expand on this sentence to state the focus of the other papers (and why they were included if the focus was not on fatigue), and how the sample sizes compared.

iii) Please consider moving page 7, lines 21-25 to the start of the Results to describe the papers that were identified.

iv) Page 7, line 27: please can you clarify here that you are referring to all included studies, and not a subgroup of primary fatigue-focused studies.

v) Page 8, line 10: the following sentences appears to be more suitable for the Discussion: “There has been a notable increase in the number of studies that measure fatigue since 2007, likely related to the inclusion of fatigue in the core outcome set following the 8th OMERACT meeting (29)…..”

vi) The Results do not contain references to the included studies. This is understandable due to the 260 papers that have been included. I wondered if there were any opportunities to highlight any references during the Results in cases of stand-out characteristics?

vii) Page 8, line 55: Again, the following sentence appears more suitable for Discussion rather than Results: “The uneven representation of men and women in the included studies may be due in part to sex imbalances in the distribution of individual conditions, but this alone does not explain the disparity.”

viii) Page 9, line 12: I suggest the definition of ‘children’ should go in the Methods not the Results, unless this decision making occurred during the analysis process, rather than being pre-defined in the Methods?

ix) I wonder if the characteristics of the participants could be better summarised in one table to show age, gender, socioeconomic status, reasons for exclusion etc rather than multiple figures

x) For any tables in which more than one answer is permitted, please could this be highlighted on the respective row/column (with an asterisk in the table and a note under the table), to enable the reader to understand why the total numbers of interventions/studies exceeds that described in the text.

xi) Could the authors expand on the theoretical basis of the interventions. I wondered if the review would identify behavioural change models. Were there differences between the theoretical basis used for adults and children?

xii) From the Appendix 5, there is reference to ‘mechanism of action’ rather than theoretical basis. I do wonder if ‘mechanism’ rather than ‘theory’ has a better fit (in answer to my query above)

Discussion

i) The authors mention activity pacing for the first time in the Discussion. Could this be introduced earlier in the Results (if not also in the Introduction if this is important)?

ii) The authors mention nutrition and exercise in the Results and Discussion at a high level of description, without stating any specific findings from the review on named supplements/types of exercise etc. Is it possible to provide some details to the manuscript from the Appendices?

iii) Page 13, line 58: please consider linking this paragraph with the PPIE section on page 10, line 20 to improve the flow of the Discussion, and generally having a section on PPIE in the Discussion may improve readability.

iv) The authors do not appear to discuss all their objectives in the Discussion, e.g. 'theoretical basis' of the interventions

v) I wondered if the authors had considered other limitations to the review, to include that there was no critical appraisal of the included studies and therefore no reporting of bias/quality. Another limitation would be the nature of analysing/synthesising the findings which was limited to presenting in tables with narrative description.

Conclusion

i) Mention of exercise to improve muscle strength and aerobic capacity first appears in the Conclusion. Please can this be added to the Discussion instead.

ii) The Conclusion should be a summary of the key points, and at present, it appears too long and lacks clear flow.

Appendices

i) Could Appendices 4 and 5 be combined for ease of reading. When reading Appendix 4, I was curious about the condition of interest.

ii) Appendix 6: if one heading is for 'fatigue as primary outcome', is the next column for 'all included studies'? The current label of 'total' is less clear.

Minor errors

Please check for typos throughout the manuscript and appendices, e.g.

Introduction, Line 48: typo on "patient" – should be "patients"

Introduction, Line 57: typo on "spondylarthritis"

Appendix 5, description of intervention for Zangi contains odd characters

---

Reviewer 2

Name Li, Bo

Affiliation Northeast Forestry University, College of Mechanical and Electrical Engineering

**Date**	**13-Sep-2025**
**COI**	**None**

1、In the Eligibility Criteria section, clearly list the specific chronic MSK conditions that are included, and state in the exclusion criteria which similar but not in-scope conditions are excluded.

2、Please clearly state in the body of the text which databases were searched, the search date range, and include in the appendix the complete search strategy for each database, including all keywords and logical operators.

3、In Data Extraction, briefly describe the extracted characteristic information about the study design, such as the number of RCTs, the number of observational studies, and the number of qualitative studies.

**Reviewer**	**3**
**Name**	**Verhagen, Arianne**
**Affiliation**	**University of Technology Sydney, Physiotherapy**
**Date**	**04-Dec-2025**
**COI**	**None**

Thank you for giving me the opportunity to review this manuscript. This manuscript concerns a scoping review on interventions for fatigue in people with chronic musculoskeletal conditions.

Generic comment

The objective is rather vague (see also some specific comments), and therefore I reviewed mainly the introduction and method section to see if I could get some clarification on the objective. This is not the case, so I would urge the authors to more clearly describe their objective.

Specific comments

1. Title. This concerns a scoping review, please state the design at the end of the title using ;.

2. Abstract. The abstract need to be a clear and concise presentation of the manuscript as a stand-alone. currently the background is focussing on effectiveness of interventions, which cannot be the focus of a scoping review, so please change. The abstract objective could be more straightforward, why is there no mention of fatigue? The abstract method should include more information. Just stating the authors follow the JBI methodology is not enough; please mention more specific choices, like selection criteria, data extraction and the data

synthesis. In the abstract result section, there is no mention at all on fatigue, and I would suggest focussing in the conclusion on an answer to the study aims.

3. Introduction.

a. The definition of ‘non-pharmacological intervention’ is vague and unclear. The definition in Wikipedia is more straightforward and provides examples: “any type of healthcare intervention which is not primarily based on medication, like . . . “.

b. Make sure the introduction clearly defines the problem, the existing evidence and which gap in the evidence this study aims to fill and why this is relevant. All this is rather unclear. Please keep in mind that a scoping review is NOT suited to define effectiveness of any intervention (see review question nr 1). What factors would you like to examine in this scoping review?

c. In addition, a scoping review, is not generating evidence, only synthesizing existing evidence. Lastly, make sure you describe an overall aim, and a primary objective and possibly a secondary (or third) objective, think about combining the current 5 objectives, and do not list the objectives.

4. Method.

a. Eligibility criteria. Please report these according to the PCC (population, concept context) framework. Make sure the exclusion criteria are not the complement of the inclusion criteria, eg including primary studies automatically means that reviews, protocols etc are not included.

b. Amendments during the screening process should be described under the heading: deviations from protocol, at the end of the method section. Combining multiple papers from one study into a single record is NOT a deviation from protocol, but mandatory in every review. One can extract different data from different papers on this single record, nevertheless.

c. I mis any information on the synthesis of the extracted data. Analysing data using narrative methods does not exist, at least there is a frequency analysis (see results, eg table 2).

5. Results. Please start here with presenting the search results and the characteristics of included studies according to the PCC framework.

VERSION 1 - AUTHOR RESPONSE

Reviewer: 1
Dr. Deborah Antcliff, Northern Care Alliance NHS Foundation Trust, Keele University Faculty of Medicine & Health Sciences

7. Thank you for the invitation to review the manuscript entitled ‘A scoping review of non-pharmacological interventions for managing fatigue across the lifespan of people living with chronic musculoskeletal conditions’. This has the potential to be an

interesting scoping review, but at present, some areas are difficult to follow and lack detail and clarification. I wondered if BMJ Open could clarify how recently they request systematic literature searches to have been undertaken and whether Nov 2023 is acceptable; and also for BMJ Open to clarify their permissive use of identical text from a published protocol in the findings paper written by the same author (see the Introduction, first four paragraphs).
Thank you for identifying the potential for our review to be of interest. As suggested, we contacted the journal to clarify how recently they request systematic literature searches to have been undertaken and the permissive use of text from a published protocol. We have provided more detail below.

Please also see major suggestions below:

Title
8. I noted the authors had changed the current title from the published protocol: “Factors influencing the outcomes of nonpharmacological interventions for managing fatigue across the lifespan of people living with musculoskeletal (MSK) conditions: a scoping review protocol”. I think the current title is perhaps more suitable since it cannot be assumed that the factors identified (e.g. sample population, theoretical basis, clinical expertise etc) directly influenced the outcomes, without undertaking specific study into each factor. However, does this change in title lessen the link between the protocol and the current paper. Do the authors wish to add ‘factors’ somewhere in the present title?
We agree that the current title is more suitable than the original title given to the protocol, as the scoping review explores the range of interventions that have been studied rather than seeking to identify specific factors relating to fatigue outcomes. Although this creates a slight discontinuity with the related protocol, we felt that it was more important that the title of the review accurately reflects its content. The published protocol has been clearly referenced in the methods.

Abstract
9. As per the PRISMA-ScR checklist, the abstract may benefit from comprising: background, objectives, eligibility criteria, sources of evidence, charting methods, results, and conclusions. At present, the abstract is missing clear eligibility criteria (e.g. types of evidence such as RCTs, cross-sectional studies etc), sources of evidence (e.g. databases etc) and charting methods.
We have amended the abstract to be more explicit about the eligibility criteria and specified the databases that we searched.

10. Objectives: in the sentence “to highlight gaps and collaborate with Patient and Public Involvement groups to identify priorities”, please add information on what priorities you are seeking to identify.
We have added information on the priorities that we are seeking to identify.

11. Results – I am unclear on the sentence: “Two hundred and sixty-eight eligible studies were found, including 72 studies primarily focused on fatigue.” From reading the start of the abstract, it would seem that a primary focus on fatigue would be part of

the inclusion criteria. Please clarify here your meaning, e.g. fatigue as a primary outcome compared with other included studies in which fatigue was a secondary outcome (if that is the case). That is, please clarify how fatigue played a role in the papers that were included in which fatigue was not the primary focus.
We would like to thank the reviewer for highlighting this. Our rationale for including studies reporting fatigue as a secondary outcome was to support a comprehensive mapping of the evidence base that captures the full range of interventions with the potential to affect fatigue. Our understanding is that this approach is standard in scoping reviews to ensure that secondary outcomes, which often provide critical context for the main research focus, are not overlooked. It also meant that we could include multi-modal studies, incidental fatigue-related findings, and interventions with holistic assessments that included fatigue.

12. Results: in the sentence "Conditions most frequently studied included fibromyalgia, rheumatoid arthritis and systemic lupus erythematous", could any percentages be provided of the proportion of studies that included the above conditions.
We acknowledge that this would be interesting, however, we didn't collect this level of detailed information. The rationale for this was due to many studies having a diverse heterogeneous MSK sample and therefore being unable to discern this level of detail..

13. Results – could a further sentence be added to report on some key findings linked to all of the aims of the review, e.g. any identified theoretical basis of the interventions, clinical competencies of those delivering interventions etc. Please also see latter comments on the use of 'theoretical basis', and whether 'mechanism' is more appropriate (as per Appendix 5).
Thank you for making this point. We have added this information to the Results.

14. Please consider providing a further sentence in the Results to link with the first sentence of the Conclusion: "Few studies explore how interventions can be combined to achieve person-centred fatigue management", since the Results do not specifically mention using singular treatments. This sentence feels out of context at present.
Thank you, as suggested, we have incorporated this point into the Results.

Introduction
15. Rationale: the first, second and fourth paragraphs contains identical text (and same references) as per the author's published protocol (with similar content also in paragraph 3) (https://bmjopen.bmj.com/content/bmjopen/14/5/e082555.full.pdf). Please check with the journal whether this exact replication of text is permissible.
Thank you for reading with such care. The advice that we received from the journal is that 'it is acceptable to base the text on your protocol, but please avoid reproducing it verbatim'. Therefore, we have re-written the paragraphs that you have identified.

16. Please check the number of people living in the UK with chronic MSK conditions since the published protocol states 10 million, but the present manuscript states 20 million. The same references have been used in both. However, these are webpages and so the number may have changed over the last 2 years. Please clarify whether the

number is 10 or 20 million and please add the date the website was visited to the References section.
Thank you for highlighting this point. The figure was taken from a report by Arthritis UK which is produced annually. The latest version was published in 2025 and states that 20.8 million people in the UK live with an MSK condition. The date that the website was accessed has been added to the reference section of the manuscript.

17. Please consider re-locating the PPI statement in the Methods rather than at the end of the Introduction.
Thank you, our PPI statement is now at the end of the Methods section as suggested and in accordance with the journal guidance.

Methods
18. Line 25-26: "A systematic search of the literature was performed in November 2023 and citation searching was completed in February 2024". Have the searches been re-run to check for any new eligible papers that have been published in the last 1-2 years?
We have re-run the searches to ensure that the evidence is up to date.

19. Page 6, line 6: the authors define their meaning of fatigue being a primary focus. However, they appear to include papers in which fatigue is not the primary focus. Please clarify how fatigue plays a role in such papers, and why they were still included in a review that focuses on fatigue.
We hope that we have addressed this point above (in the comment relating to the Results). To clarify, we included studies in which fatigue is a secondary outcome because they can provide important contextual information and ensure the scoping review captures the breadth of the evidence base.

20. Please can the authors expand on the analysis section of the Methods, beyond stating narrative methods were used (e.g. actual analysis such as coding/thematic analysis, methods to present the results, methods to summarise/synthesise the evidence). If these stages did not occur, please state as such and explain why.
Thank you for raising this point. Our approach was informed by the JBI Evidence Synthesis 'Recommendations for the extraction, analysis, and presentation of results in scoping reviews' (reference 28). Our analysis focused on descriptive approaches, including frequency counts and basic qualitative content analysis rather than interpretive or advanced synthesis methods. We decided that a thematic analysis was not appropriate for our scoping review because we were not exploring people's experiences and/or their sense making processes and that level of data interpretation does not align with our scoping aims. Instead, we familiarised ourselves with the data, used open coding, and organised these codes into categories. We took care to avoid reinterpretation or claims about effectiveness due to the absence of critical appraisal. We chose to present our findings using a combination of tables, narrative summaries, and visual formats, such as charts and diagrams, to communicate how we had mapped the evidence. As suggested, we have expanded the analysis section.

21 Please can the authors state they have reported the review as per the PRISMA-ScR checklist in the Methods.
We have included this statement in the Methods section as suggested.

22. As per the PRISMA-ScR checklist, please can the authors add detail to the Methods to "list and define all variables for which data were sought and any assumptions and simplifications made." For example, the authors state in the objectives they will explore papers for theoretical basis, which is not mentioned in the Methods.
Thank you for highlighting this omission. We have now included in the main text an overview of the variables for which data were sought, ensuring that our search data related to any theoretical basis for interventions is stated. Due to the large number of variables included we have not given a full description of this within the text and have instead summarised the areas of interest and signposted to both the template in the published protocol and the amended template, which is included as Appendix 2.

23. Were any critical appraisals undertaken of the included studies? If not, please can this be stated as a limitation in the Discussion.
We can confirm that we did not undertake critical appraisals of the included studies, as the JBI states that critical appraisal is generally not required because scoping reviews aim to map evidence, not assess its quality. However, we have added this as a limitation in the Discussion.

24. Please signpost to the PRISMA flowchart from the Results not the Methods.
Thank you, this has now been amended

Results
25. Please commence the results with the details as per the PRISMA flowchart to report on number of studies identified and included in the review. I am unclear as to which studies were included or excluded. Specifically, the authors describe papers in which the primary focus is on fatigue. If the primary focus was not on fatigue, were the papers still included in the review? Please clarify your meaning of fatigue being a primary focus, e.g. as a primary outcome, compared with other included studies in which fatigue was a secondary outcome (if I have interpreted this is correctly). See earlier comment on this in the Methods.
Thank you for highlighting this. We distinguished between fatigue as a primary and secondary outcome when we reported our findings to provide a nuanced map of the evidence base. This distinction allows us to differentiate between interventions specifically developed to target fatigue and those where fatigue is captured as a co-existing symptom, exploratory variable or incidental finding. Including fatigue as both a primary and secondary outcome ensures the scoping review captures the full breadth of the available data, while the distinction helps show the extent to which fatigue is treated as a clinical priority. The PRISMA flow diagram has been updated to include the updated searches and enhance clarity.

26. Page 7, line 21: "the studies which focused primarily on fatigue had smaller numbers of participants." Please see point Results, comment i) about clarifying which papers you have included as being focused on fatigue. Please also expand on this

sentence to state the focus of the other papers (and why they were included if the focus was not on fatigue), and how the sample sizes compared.
Thank you for highlighting this point. We have amended this paragraph for clarity, including our rationale for including papers where fatigue was not the focus of the studied intervention (see above comment).

27. Please consider moving page 7, lines 21-25 to the start of the Results to describe the papers that were identified.
Thank you, we have amended this paragraph to reflect the updated searches and moved it to the beginning of the Results to improve the overall flow of this section.

28. Page 7, line 27: please can you clarify here that you are referring to all included studies, and not a subgroup of primary fatigue-focused studies.
Thank you, we have amended this line for clarity

29. Page 8, line 10: the following sentences appears to be more suitable for the Discussion: "There has been a notable increase in the number of studies that measure fatigue since 2007, likely related to the inclusion of fatigue in the core outcome set following the 8th OMERACT meeting (29)….."
Thank you, we have moved this to the Discussion.

30. The Results do not contain references to the included studies. This is understandable due to the 260 papers that have been included. I wondered if there were any opportunities to highlight any references during the Results in cases of stand-out characteristics?
Thank you for this suggestion. Due to the breadth of the review we have chosen to focus on the shared characteristics of the included papers and the gaps revealed when mapping the evidence rather than to focus on individual papers. We have provided details of the individual interventions (Appendix 4) for all included studies as supplementary material to give readers the opportunity to explore our data in greater depth if desired.

31. Page 8, line 55: Again, the following sentence appears more suitable for Discussion rather than Results: "The uneven representation of men and women in the included studies may be due in part to sex imbalances in the distribution of individual conditions, but this alone does not explain the disparity.
Thank you, this statement has been moved to the Discussion section as suggested.

32. Page 9, line 12: I suggest the definition of 'children' should go in the Methods not the Results, unless this decision making occurred during the analysis process, rather than being pre-defined in the Methods?
Thank you for this suggestion, the statement has been moved to the Methods

33. I wonder if the characteristics of the participants could be better summarised in one table to show age, gender, socioeconomic status, reasons for exclusion etc rather than multiple figures

Thank you for this suggestion. As this review includes such a large amount of data it is not possible to represent it all in a single table. We have attempted to represent the data as fully and clearly as possible within the limitations of the presentation style and we will make data available upon reasonable request to any interested parties.

34. For any tables in which more than one answer is permitted, please could this be highlighted on the respective row/column (with an asterisk in the table and a note under the table), to enable the reader to understand why the total numbers of interventions/studies exceeds that described in the text.
Thank you for this suggestion, explanatory notes have been added where appropriate.

35. Could the authors expand on the theoretical basis of the interventions. I wondered if the review would identify behavioural change models. Were there differences between the theoretical basis used for adults and children?
We agree that this is an interesting point to understand. To avoid reinterpreting or overinterpreting data, we have presented information on the mechanism category (the theoretical basis and/or mechanisms of change) as it has been reported in the studies (see Appendix 5). One of our main findings is that comparatively few studies explained how the interventions were hypothesised to bring about change in any detail. In comparison to adults, the studies relating to children and young people almost always stated a physical theoretical basis for their intervention such as reducing fatigue by increasing strength and/or aerobic capacity.

36. From the Appendix 5, there is reference to 'mechanism of action' rather than theoretical basis. I do wonder if 'mechanism' rather than 'theory' has a better fit (in answer to my query above)
Thank you for raising this interesting point. We looked for information on theory to explain why an intervention could lead to change and information on mechanisms of action to explain how the intervention could lead to change. We think that including both means we capture the most evidence, rather than one being a better fit than the other. However, this distinction was not made in many studies and the detail provided was minimal. Therefore, in Table 3, we used the term 'Mechanism category' to capture the type of process or underlying reason why an intervention might produce a particular outcome. We realise that the use of related, sometimes overlapping terms, is confusing. In response, we have changed Objective 3 to Mechanism category.

Discussion
37. The authors mention activity pacing for the first time in the Discussion. Could this be introduced earlier in the Results (if not also in the Introduction if this is important)?
As suggested, we have made changes to the text on psychological mechanisms in the Results and mentioned activity pacing.

38. The authors mention nutrition and exercise in the Results and Discussion at a high level of description, without stating any specific findings from the review on named supplements/types of exercise etc. Is it possible to provide some details to the manuscript from the Appendices?

In the discussion of physical activity interventions, the number of included studies and the fact that we have not attempted to determine the efficacy of interventions make it impractical to highlight the findings from specific studies. However, we have provided a brief description of each intervention in Appendix 4 to allow readers to explore areas of interest in greater depth. When discussing nutrition, the key point is the discrepancy between the interest shown in this topic by PPIE contributors and the amount of available evidence. It did not feel appropriate to include specific references to nutritional supplements or regimes as the efficacy of these was not assessed as part of this review.

39. Page 13, line 58: please consider linking this paragraph with the PPIE section on page 10, line 20 to improve the flow of the Discussion, and generally having a section on PPIE in the Discussion may improve readability.
Thank you for this suggestion, we have amended the manuscript to reflect this.

40. The authors do not appear to discuss all their objectives in the Discussion, e.g. 'theoretical basis' of the interventions
Thank you for this suggestion, we have expanded the Discussion to include all review objectives.

41. I wondered if the authors had considered other limitations to the review, to include that there was no critical appraisal of the included studies and therefore no reporting of bias/quality. Another limitation would be the nature of analysing/synthesising the findings which was limited to presenting in tables with narrative description.
As suggested, we have added the lack of critical appraisal and lack of detail about individual interventions as limitations.

Conclusion
42. Mention of exercise to improve muscle strength and aerobic capacity first appears in the Conclusion. Please can this be added to the Discussion instead.
This has been amended as suggested.

43. The Conclusion should be a summary of the key points, and at present, it appears too long and lacks clear flow.
Amendments have been made to this section to improve clarity.

Appendices
44. Could Appendices 4 and 5 be combined for ease of reading. When reading Appendix 4, I was curious about the condition of interest.
The amount of data is too large to easily produce in a single table; however, we have added the MSK condition of interest to Appendix 4 for clarity.

45. Appendix 6: if one heading is for 'fatigue as primary outcome', is the next column for 'all included studies'? The current label of 'total' is less clear.
Thank you for the suggestion, this has been amended.

46. Minor errors

Please check for typos throughout the manuscript and appendices, e.g.
Introduction, Line 48: typo on “patient” – should be “patients”
Introduction, Line 57: typo on “spondylarthritis”
Appendix 5, description of intervention for Zangi contains odd characters
Thank you for making us aware of these errors, further reviewing of the manuscript for typos has been undertaken by the team.

Reviewer: 2
Dr. Bo Li, Northeast Forestry University

47. In the Eligibility Criteria section, clearly list the specific chronic MSK conditions that are included, and state in the exclusion criteria which similar but not in-scope conditions are excluded.
We appreciate this point. However, as the list of eligible conditions is extensive, we have included this within the full search strategy, presented in Appendix 1.

48、Please clearly state in the body of the text which databases were searched, the search date range, and include in the appendix the complete search strategy for each database, including all keywords and logical operators.
Thank you, we have added the names of the databases that we searched to the body of the text. The full search strategy is included in Appendix 1.

49. In Data Extraction, briefly describe the extracted characteristic information about the study design, such as the number of RCTs, the number of observational studies, and the number of qualitative studies.
Data on the type of study was collected however, we only differentiated between whether a study was qualitative, quantitative or included mixed methods. The number of different types of studies included is discussed in the Results within Review Objective 4.

Reviewer: 3
Prof. Arianne Verhagen, University of Technology Sydney

Thank you for giving me the opportunity to review this manuscript. This manuscript concerns a scoping review on interventions for fatigue in people with chronic musculoskeletal conditions.

Generic comment
50. The objective is rather vague (see also some specific comments), and therefore I reviewed mainly the introduction and method section to see if I could get some clarification on the objective. This is not the case, so I would urge the authors to more clearly describe their objective.
Thank you for taking the time to review our manuscript. Below, we have responded to each of your points, and we hope that this has helped clarify the work.

Specific comments

51. Title. This concerns a scoping review, please state the design at the end of the title using ;.
As suggested, we have re-ordered the wording in the title.

52. Abstract. The abstract need to be a clear and concise presentation of the manuscript as a stand-alone. currently the background is focussing on effectiveness of interventions, which cannot be the focus of a scoping review, so please change. The abstract objective could be more straightforward, why is there no mention of fatigue? The abstract method should include more information. Just stating the authors follow the JBI methodology is not enough; please mention more specific choices, like selection criteria, data extraction and the data synthesis. In the abstract result section, there is no mention at all on fatigue, and I would suggest focussing in the conclusion on an answer to the study aims.
Thank you, we have made the following changes to the abstract:

- We have removed the wording relating to intervention effectiveness from the Background in the abstract.
- We have re-worded the objective to highlight that the focus is on fatigue in MSK conditions.
- We have added more detail to the Methods section.
- We have mentioned fatigue at the start of the Results section.

Introduction.
53. The definition of 'non-pharmacological intervention' is vague and unclear. The definition in Wikipedia is more straightforward and provides examples: "any type of healthcare intervention which is not primarily based on medication, like . . . ".
We appreciate that the definition we used is complex. As suggested, we have simplified the definition and provided supporting references.

- *Ninot, G. (2022). Non-Pharmacological Interventions for the Management of Chronic Health Conditions and Non-Communicable Diseases. International Journal of Environmental Research and Public Health, 19(14), 8443*
- *https://www.jamda.com/article/S1525-8610(24)00728-X/fulltext*

54. Make sure the introduction clearly defines the problem, the existing evidence and which gap in the evidence this study aims to fill and why this is relevant. All this is rather unclear. Please keep in mind that a scoping review is NOT suited to define effectiveness of any intervention (see review question nr 1). What factors would you like to examine in this scoping review?
Thank you for these reflections. We have added more detail about the problem, explaining that fatigue is a patient priority, but interventions are not part of standard care (reference 23). To clarify that our scoping review is not looking at effectiveness, we have changed the wording of the first objective, from 'evidence for' (which might imply effectiveness) to 'type of' intervention.

55. In addition, a scoping review, is not generating evidence, only synthesizing existing evidence. Lastly, make sure you describe an overall aim, and a primary objective and possibly a secondary (or third) objective, think about combining the current 5 objectives, and do not list the objectives.

We agree that the rationale of a scoping review is to synthesise existing evidence, rather than to generate it. We have tweaked the wording to clarify our two overall aims. However, we prefer not to rank the objectives (primary, secondary etc.) or to combine them, as we want to focus on breadth.

Method.

56. Eligibility criteria. Please report these according to the PCC (population, concept context) framework. Make sure the exclusion criteria are not the complement of the inclusion criteria, eg including primary studies automatically means that reviews, protocols etc are not included.

Thank you for this suggestion. As the eligibility criteria were published in the review protocol we have chosen to not make this amendment, to avoid creating a false impression that the eligibility criteria were changed.

57. Amendments during the screening process should be described under the heading: deviations from protocol, at the end of the method section. Combining multiple papers from one study into a single record is NOT a deviation from protocol, but mandatory in every review. One can extract different data from different papers on this single record, nevertheless.

Thank you for this suggestion, these statements have been moved to the end of the Methods and labelled as suggested.

58. I mis any information on the synthesis of the extracted data. Analysing data using narrative methods does not exist, at least there is a frequency analysis (see results, eg table 2).

Thank you for highlighting that this is not clear. We have added detail to the section on data charting. Please see the response above to comment 22.

59. Results. Please start here with presenting the search results and the characteristics of included studies according to the PCC framework.

Please see the response to comment 25 above.

VERSION 2 - REVIEW

Reviewer 1

Name Antcliff, Deborah

Affiliation Northern Care Alliance NHS Foundation Trust, Bury Integrated Pain Service

Date 17-Apr-2026

COI

Thank you for responding to the comments from the reviewers and revising the manuscript entitled ‘Non-pharmacological interventions for managing fatigue across the lifespan of people living with chronic musculoskeletal conditions; a scoping review’.

Overall, the manuscript has improved following these revisions. It is not underestimated the amount of work that is required to update the searches, especially in such a broad scoping review. Re-running the searches has been beneficial, since a significant number of papers have been added.

There are a few places which are difficult to follow and in which further clarity would be beneficial. I have made some major and minor suggestions for your consideration as below:

Major suggestions

Abstract

1. Thank you for adding detail to the Abstract in keeping with the PRISMA-ScR checklist. It may also be beneficial for the reader to see the search date, so that the search is clearly between 1st Jan 2007-and [insert day] Feb 2026.

2. The Abstract currently does not contain clear information on ‘charting methods’ as per the PRISMA-ScR checklist. Could a few words be added to summarise how data were charted?

Aims

3. You have changed your aims to mention ‘mechanism category’, but use the term ‘theoretical basis’ in the Abstract and Methods. Should these terms align for consistency throughout the manuscript? If you are talking about two different concepts, please clarify in the manuscript what you mean by mechanism or theoretical basis.

4. Reviewer 3 highlighted that the scoping review would not generate evidence, yet this is still stated as one of the aims.

Methods

5. Please add the search day onto the re-run search date, i.e. [insert day] Feb 2026.

Discussion

6. I am finding the Discussion hard to follow. I wondered if the lead author wanted to implement the Track changes comment by JP35R34 and open the Discussion by returning to the original aims of the review. The aims could then be used to structure the Discussion, e.g. exploring the type of intervention, then characteristics, then mechanism etc. At present, the Discussion appears to mix up paragraphs that discuss mechanisms, participant samples/characteristics, PPIE and gaps in the literature, which limits the flow of this section.

7. In response to comment #39, the authors have moved a paragraph on PPIE to the Discussion. I am sorry if my comment was not clear. The Discussion now appears to now contain some methods regarding a workshop that was undertaken after the review. Has this workshop been mentioned before in the appropriate Methods, Results or PPI section? If not,

please state what happened in the appropriate section of the manuscript (e.g. a sentence in the PPI section of the Methods to state workshops were held after the review to help interpret the results, e.g. on page 6, line 35), and then use the Discussion to consider the implications/findings from this.

8. Figures

PRIMSA flow chart: for the reasons of exclusion, I wonder if it is more appropriate to write "not MSK population" rather than "wrong population" (since the population/intervention would have been 'right' for that particular study).

Minor suggestions

Results

1. Typo on the note under Table 3: "the total number of included studies is few than the combined individual counts across intervention type where an intervention has included multiple components". "Few" needs to be "fewer".

2. Table 3: Would it be helpful and relevant to add a bottom row to show 'totals'.

**Reviewer**	**3**
**Name**	**Verhagen, Arianne**
**Affiliation**	**University of Technology Sydney, Physiotherapy**
**Date**	**13-Apr-2026**
**COI**	

Thank you for giving me the opportunity to re-review this manuscript. The manuscript has improved majorly based on the comments, but some things remain. More specifically, I understand authors will not always follow all comments, but a proper rationale is not always provided.

The manuscript is still overy long and some long sentences create a vagueness that is not necessary. In addition there is a lot of repetition too. Revising this manuscript to be more concise and specific would be recommended.

1. Abstract.

a) Although the abstract is improved, there still is some information missing, things are unclear or vague.

b) For instance, the objective is now a sentence of over 4 lines long and still too vague for me. I suggest a rephrasing of the objective like the following: "To create an overview of current knowledge on the description (like theoretical basis, patient characterisitcs, caregiver competenties) of non-pharmacological interventions for fatige in musculoskeletal

conditions. The findings help to identify priorities for research and practice in collaboration with Patient and Public Involvement groups.“

c) For the method more or less the same comment exist, it‘s vague, and still lacking informaiton. May I suggest the following: „This scoping review followed the Joanna Briggs Institute methodology. Peer-reviewed primary English language research studies are eligible that describe a non-pharmacological intervention aiming to reducing fatigue in musculoskeletal conditions. We searched seven databases from 1st January 2007 to February 2026. We extracted . . . . and performed a descriptive analysis.”

d) I do not see the rationale for distinguishing between studies that had fatigue as their primary focus or their secondary focus (see also comment 25 and 26). This is not relevant for the current study as none of the research questions is related to this issue. Therefore, all mention on studies that have a primary focus on fatigue can be deleted in my humble opinion. Please report results on all research questions in the abstract, result section. My suggestion for rephrasing the result section would be as follows: “Out of 9527 references found, 337 studies were eligible, of which ?? trials, ??cohorts studies . . Conditions most frequently studied included fibromyalgia, rheumatoid arthritis and systemic lupus erythematous. Physical activity was the commonest type of intervention, most participants were adults and approximately 82% were female. Most (??%) studies reported single component interventions, and few included a theoretical basis or the clinical competencies required to deliver them. Outcomes reported entail: .”

e) Lastly, the conclusion is still not a clear answer to the study questions.

2. Introduction.

a. I am still not happy with the 5 bullet point research questions, and I do not understand the answer of the authors. I leave this issue with the editor. Using bullet point in a research manuscript is generally not recommended

3. Method.

a. Eligibility criteria. My initial comments was to make sure the exclusion criteria are not the complement of the inclusion criteria, eg including primary studies automatically means that reviews, protocols etc are not included. The fact this can be differently reported compared to the protocol, does not automatically mean creating a false impression.

4. Results.

a. In presenting the results, please reconsider the focus on fatigue being the primary focus of the study or not. This seems to me irrelevant information as it does not answer any of the current research questions

b. Subheadings like: “Review objective1: Explore the type of existing interventions for MSK fatigue.” are overly long. I would suggest shortening this to: Type of interventions. The

authors do not need to refer to the specific research question, and do not have to repeat that this concerns fatigue in MSK.

VERSION 2 - AUTHOR RESPONSE

Reviewer: 3
Prof. Arianne Verhagen, University of Technology Sydney
Comments to the Author:

Thank you for giving me the opportunity to re-review this manuscript. The manuscript has improved majorly based on the comments, but some things remain. More specifically, I understand authors will not always follow all comments, but a proper rationale is not always provided.

Thank you for taking the time to re-review. We are pleased to see that you think the manuscript has improved majorly. We hope that we can provide more rationale where you find it lacking.

The manuscript is still overly long and some long sentences create a vagueness that is not necessary. In addition there is a lot of repetition too. Revising this manuscript to be more concise and specific would be recommended.

1. Abstract.
a) Although the abstract is improved, there still is some information missing, things are unclear or vague.
b) For instance, the objective is now a sentence of over 4 lines long and still too vague for me. I suggest a rephrasing of the objective like the following: “To create an overview of current knowledge on the description (like theoretical basis, patient characteristics, caregiver competencies) of non-pharmacological interventions for fatigue in musculoskeletal conditions. The findings help to identify priorities for research and practice in collaboration with Patient and Public Involvement groups.“
As you suggest, we have split the single, long sentence into two shorter sentences. We have also revised the abstract formatting as requested by the Editor.

c) For the method more or less the same comment exist, it‘s vague, and still lacking information. May I suggest the following: „This scoping review followed the Joanna Briggs Institute methodology. Peer-reviewed primary English language research studies are eligible that describe a non-pharmacological intervention aiming to reducing fatigue in musculoskeletal conditions. We searched seven databases from 1st January 2007 to February 2026. We extracted . . . . and performed a descriptive analysis.”

Thank you for the suggested wording. As above, we have made changes in line with your suggestions and the Editor’s requirements.

d) I do not see the rationale for distinguishing between studies that had fatigue as their primary focus or their secondary focus (see also comment 25 and 26). This is not relevant for the current study as none of the research questions is related to this issue.

Therefore, all mention on studies that have a primary focus on fatigue can be deleted in my humble opinion. Please report results on all research questions in the abstract, result section. My suggestion for rephrasing the result section would be as follows: "Out of 9527 references found, 337 studies were eligible, of which ?? trials, ??cohorts studies . . Conditions most frequently studied included fibromyalgia, rheumatoid arthritis and systemic lupus erythematous. Physical activity was the commonest type of intervention, most participants were adults and approximately 82% were female. Most (??%) studies reported single component interventions, and few included a theoretical basis or the clinical competencies required to deliver them. Outcomes reported entail: ."

As you suggest, we have removed the distinction between studies that had fatigue as their primary focus or secondary focus from the Results section of the Abstract. The revised Results section now includes much of the information that you suggest. We have not distinguished between trials, cohort studies etc. because our focus was on scoping the existence of primary evidence rather than evaluating specific study designs.

e) Lastly, the conclusion is still not a clear answer to the study questions.

We have re-written the conclusion to more clearly answer the study questions.

2. Introduction.
a. I am still not happy with the 5 bullet point research questions, and I do not understand the answer of the authors. I leave this issue with the editor. Using bullet point in a research manuscript is generally not recommended

We have re-formatted the aims and objectives and removed the bullet points.

3. Method.
a. Eligibility criteria. My initial comments was to make sure the exclusion criteria are not the complement of the inclusion criteria, eg including primary studies automatically means that reviews, protocols etc are not included. The fact this can be differently reported compared to the protocol, does not automatically mean creating a false impression.
4. Results.
a. In presenting the results, please *reconsider* the focus on fatigue being the primary focus of the study or not. This seems to me irrelevant information as it does not answer any of the current research questions

We appreciate that the focus on fatigue as the primary outcome was not one of the questions. However, we think that it is important to report that while fatigue is a patient priority and measurement is recommended, it is less often a treatment target. Beckers et al., 2023 (ref. 18) also make the distinction between the focus of fatigue as a primary or secondary outcome in their scoping review. We have added a sentence in the Discussion to explain this "*However, fatigue was often a secondary outcome rather than the treatment target"*.

b. Subheadings like: "Review objective1: Explore the type of existing interventions for MSK fatigue." are overly long. I would suggest shortening this to: Type of interventions. The authors do not need to refer to the specific research question, and do not have to repeat that this concerns fatigue in MSK.

Thank you. In line with your suggestion, we have shortened the Review objective sub-headings.

Reviewer: 1
Dr. Deborah Antcliff, Northern Care Alliance NHS Foundation Trust, Keele University Faculty of Medicine & Health Sciences
Comments to the Author:

Thank you for responding to the comments from the reviewers and revising the manuscript entitled 'Non-pharmacological interventions for managing fatigue across the lifespan of people living with chronic musculoskeletal conditions; a scoping review'.

Overall, the manuscript has improved following these revisions. It is not underestimated the amount of work that is required to update the searches, especially in such a broad scoping review. Re-running the searches has been beneficial, since a significant number of papers have been added.

Thank you for acknowledging the amount of work we have undertaken. We agree that the review is strengthened as a result.

There are a few places which are difficult to follow and in which further clarity would be beneficial. I have made some major and minor suggestions for your consideration as below:

Major suggestions
Abstract
1. Thank you for adding detail to the Abstract in keeping with the PRISMA-ScR checklist. It may also be beneficial for the reader to see the search date, so that the search is clearly between 1st Jan 2007-and [insert day] Feb 2026.

Thank you. We have re-formatted the Abstract based on reviewers' suggestions and the Editor's requirements.

2. The Abstract currently does not contain clear information on 'charting methods' as per the PRISMA-ScR checklist. Could a few words be added to summarise how data were charted?

We have added 'Data were charted using the protocol template' to the Abstract.

Aims
3. You have changed your aims to mention 'mechanism category', but use the term 'theoretical basis' in the Abstract and Methods. Should these terms align for

consistency throughout the manuscript? If you are talking about two different concepts, please clarify in the manuscript what you mean by mechanism or theoretical basis.

Thank you for highlighting this. We agree that our use of both theoretical basis and mechanism category is potentially confusing. In Review objective 3, we have clarified that the theoretical basis explains the conceptual rationale about why the intervention should work, while the mechanisms of action describe the specific processes through which the intervention produces its effects, linking theory to observable outcomes. We have made substantial changes to address this.

4. Reviewer 3 highlighted that the scoping review would not generate evidence, yet this is still stated as one of the aims.

Thank you, we appreciate that we have not been clear. We have revised the aims from generate evidence to report existing evidence.

Methods

5. Please add the search day onto the re-run search date, i.e. [insert day] Feb 2026.

We have added this.

Discussion

6. I am finding the Discussion hard to follow. I wondered if the lead author wanted to implement the Track changes comment by JP35R34 and open the Discussion by returning to the original aims of the review. The aims could then be used to structure the Discussion, e.g. exploring the type of intervention, then characteristics, then mechanism etc. At present, the Discussion appears to mix up paragraphs that discuss mechanisms, participant samples/characteristics, PPIE and gaps in the literature, which limits the flow of this section.

We have re-structured the Discussion section. We hope this has improved the flow.

7. In response to comment #39, the authors have moved a paragraph on PPIE to the Discussion. I am sorry if my comment was not clear. The Discussion now appears to now contain some methods regarding a workshop that was undertaken after the review. Has this workshop been mentioned before in the appropriate Methods, Results or PPI section? If not, please state what happened in the appropriate section of the manuscript (e.g. a sentence in the PPI section of the Methods to state workshops were held after the review to help interpret the results, e.g. on page 6, line 35), and then use the Discussion to consider the implications/findings from this.

As you suggest, we have described the PPIE workshops in the Methods section and used the Discussion to consider how PPIE shaped the review overall.

8. Figures

PRIMSA flow chart: for the reasons of exclusion, I wonder if it is more appropriate to write "not MSK population" rather than "wrong population" (since the population/intervention would have been 'right' for that particular study).

This has been re-labelled as you have suggested.

Minor suggestions
Results
1. Typo on the note under Table 3: "the total number of included studies is few than the combined individual counts across intervention type where an intervention has included multiple components". "Few" needs to be "fewer".

Thank you for highlighting the typo. We have corrected it.

2. Table 3: Would it be helpful and relevant to add a bottom row to show 'totals'.

Thank you for the suggestion. We decided not to add a bottom row, as we have included total numbers of interventions in Table 1.

VERSION 3 - REVIEW

Reviewer 1
Name Antcliff, Deborah
Affiliation Northern Care Alliance NHS Foundation Trust, Bury Integrated Pain Service
Date 23-Jun-2026
COI

Thank you for submitting further amendments to the manuscript entitled: 'Non-pharmacological interventions for managing fatigue across the lifespan of people living with chronic musculoskeletal conditions; a scoping review'. Following these amendments, the manuscript is much improved. In particular, the Discussion has a greater sense of flow and readability.

I have some very minor comments for the authors' consideration:

Abstract

1) In response to my previous comment asking for a brief description of the charting approach, the authors now state: "Data were charted using the protocol template". From this sentence, I am sorry that I still do not have a better understanding of the charting method (without needing to read the protocol). Please could the authors briefly state how the data were charted, or provide an example (e.g. data were charted in tables or categories

etc…. according to type of study/population/type of intervention/level of supervision of the intervention…), as appropriate?

2) Would the authors consider adding to the 'Design' subheading to state this is a 'scoping review' (and perhaps just use the term 'review' as it appears in the 'Objectives' subheading.

Methods

3) I am still unclear about the charting methods (beyond the adaptations suggested by PPIE). A couple of sentences to give examples of how charting was undertaken would be beneficial.

**Reviewer**	**3**
**Name**	**Verhagen, Arianne**
**Affiliation**	**University of Technology Sydney, Physiotherapy**
**Date**	**14-Jun-2026**
**COI**	

Thank you for giving me the opportunity to re-review this manuscript. The manuscript has improved again, but some minor things remain.

1. Abstract. Please state under the subheading 'design' the design, which is a scoping review. In addition, change 'narrative synthesis of results' into 'descriptive analysis of the results' and add numbers or percentages after 'most'.

2. Method. The section related to amendments to the protocol (now under study selection) should be placed under the heading: 'Deviations from protocol' at the end of the method section (no bullet points please).

3. Results.

a. I repeat my comment on the headings: "Subheadings like: "Review objective1: Explore the type of existing interventions for MSK fatigue." are overly long. I would suggest shortening this to: Type of interventions. The authors do not need to refer to the specific research question, and do not have to repeat that this concerns fatigue in MSK."

b. I also repeat my comment on the use of bullet points. Please refrain from using bullet points at all except in tables.

VERSION 3 - AUTHOR RESPONSE

Reviewer: 3
Prof. Arianne Verhagen, University of Technology Sydney
Comments to the Author:

Thank you for giving me the opportunity to re-review this manuscript. The manuscript has improved again, but some minor things remain.

1. Abstract. Please state under the subheading 'design' the design, which is a scoping review. In addition, change 'narrative synthesis of results' into 'descriptive analysis of the results' and add numbers or percentages after 'most'.

Thank you for your suggestions, these amendments have been made.

2. Method. The section related to amendments to the protocol (now under study selection) should be placed under the heading: 'Deviations from protocol' at the end of the method section (no bullet points please).

All deviations from the published protocol are now placed in the appropriate section.

3. Results.
a. I repeat my comment on the headings: "Subheadings like: "Review objective1: Explore the type of existing interventions for MSK fatigue." are overly long. I would suggest shortening this to: Type of interventions. The authors do not need to refer to the specific research question, and do not have to repeat that this concerns fatigue in MSK."

Thank you for your suggestion – this title was shortened to "Review objective 1: Explore the type of existing interventions" in the last revision.

b. I also repeat my comment on the use of bullet points. Please refrain from using bullet points at all except in tables.
Thank you for your suggestion, the definitions of the intervention types have been presented as a table

Reviewer: 1
Dr. Deborah Antcliff, Northern Care Alliance NHS Foundation Trust, Keele University Faculty of Medicine & Health Sciences
Comments to the Author:

Thank you for submitting further amendments to the manuscript entitled: 'Non-pharmacological interventions for managing fatigue across the lifespan of people living with chronic musculoskeletal conditions; a scoping review'. Following these amendments, the manuscript is much improved. In particular, the Discussion has a greater sense of flow and readability.

I have some very minor comments for the authors' consideration:

Abstract

1) In response to my previous comment asking for a brief description of the charting approach, the authors now state: “Data were charted using the protocol template”. From this sentence, I am sorry that I still do not have a better understanding of the charting method (without needing to read the protocol). Please could the authors briefly state how the data were charted, or provide an example (e.g. data were charted in tables or categories etc.... according to type of study/population/type of intervention/level of supervision of the intervention...), as appropriate?

Thank you for your suggestion, I have amended this paragraph to give additional information.

2) Would the authors consider adding to the ‘Design’ subheading to state this is a ‘scoping review’ (and perhaps just use the term ‘review’ as it appears in the ‘Objectives’ subheading.

Thank you for these suggestions, the manuscript has been amended accordingly

Methods

3) I am still unclear about the charting methods (beyond the adaptations suggested by PPIE). A couple of sentences to give examples of how charting was undertaken would be beneficial.

Thank you for your suggestion, I have added additional information to this section including a link to the charting template.
