# Supplementary material for: Non-pharmacological interventions for managing fatigue across the lifespan of people living with chronic musculoskeletal conditions: a scoping review
Source: BMJ Open. 2026 Aug 4;16(8):e108929. doi: 10.1136/bmjopen-2025-108929 (PMC13448569; doi:10.1136/bmjopen-2025-108929)
Supplement: Supplementary file 4 [file bmjopen-16-8-s003.docx]

Appendix 3. GRIPP2 Short form reporting checklist

| Section and topic | Item | Reported on page No |
| --- | --- | --- |
| 1: Aim | Report the aim of PPI in the study | 5 |
| 2: Methods | Provide a clear description of the methods used for PPI in the study | 3-4 |
| 3: Study results | Outcomes—Report the results of PPI in the study, including both positive and negative outcomes | 5 |
| 4: Discussion and conclusions | Outcomes—Comment on the extent to which PPI influenced the study overall. Describe positive and negative effects | 10-12 |
| 5: Reflections/critical perspective | Comment critically on the study, reflecting on the things that went well and those that did not, so others can learn from this experience | 12 |
